# Roles of Cholesteryl-α-Glucoside Transferase and Cholesteryl Glucosides in Maintenance of Helicobacter pylori Morphology, Cell Wall Integrity, and Resistance to Antibiotics

**DOI:** 10.1128/mBio.01523-18

**Published:** 2018-11-27

**Authors:** Majjid A. Qaria, Naveen Kumar, Arif Hussain, Shamsul Qumar, Sankara N. Doddam, Ludovico P. Sepe, Niyaz Ahmed

**Affiliations:** a Pathogen Biology Laboratory, Department of Biotechnology and Bioinformatics, University of Hyderabad, Hyderabad, India; b Department of Molecular Biology, Max-Planck Institute for Infection Biology, Berlin, Germany; c International Centre for Diarrhoeal Disease Research, Bangladesh (icddr,b), Dhaka, Bangladesh; KUMC; Oita University Faculty of Medicine; University of Malaya; Oxford University Clinical Research Unit; KIIT School of Biotechnology

**Keywords:** *H. pylori*, cholesteryl glucosides, morphology, membrane permeability, antibiotic susceptibility, biofilm formation, *Helicobacter pylori*, antibiotic resistance, cell wall integrity

## Abstract

Infection of the human stomach caused by Helicobacter pylori is very common, as the pathogen colonizes more than half of the world’s population. It is associated with varied outcomes of infection, such as peptic ulcer disease, gastric ulcers, and mucosa-associated lymphoid tissue lymphoma, and is generally considered a risk factor for the development of gastric adenocarcinoma. Cholesteryl glucosides (CGs) constitute a vital component of the cell wall of H. pylori and contribute to its pathogenicity and virulence. The *hp0421* gene, which encodes cholesteryl-α-glucoside transferase (CGT), appears critical for the enzymatic function of integrating unique CGs into the cell wall of H. pylori, and deletion of this gene leads to depletion of CGs and their variants. Herein, we report that the deletion of *hp0421* and consequent deficiency of cholesterol alter the morphology, shape, and cell wall composition of H. pylori cells, as demonstrated by high-resolution confocal microscopy and flow cytometry analyses of two different type strains of H. pylori, their isogenic knockouts as well as a reconstituted strain. Moreover, measurement of ethidium bromide (EtBr) influx by flow cytometry showed that lack of CGs increased cell wall permeability. Antimicrobial susceptibility testing revealed that the *hp0421* isogenic knockout strains (*Hp26695Δ421* and *Hp76Δ421*) were sensitive to antibiotics, such as fosfomycin, polymyxin B, colistin, tetracycline, and ciprofloxacin, in contrast to the wild-type strains that were resistant to the above antibiotics and tended to form denser biofilms. Lipid profile analysis of both *Hp76* and *Hp76Δ421* strains showed an aberrant profile of lipopolysaccharides (LPS) in the *Hp76Δ421* strain. Taken together, we herein provide a set of mechanistic evidences to demonstrate that CGs play critical roles in the maintenance of the typical spiral morphology of H. pylori and its cell wall integrity, and any alteration in CG content affects the characteristic morphological features and renders the H. pylori susceptible to various antibiotics.

## Abstract (precis)

Helicobacter pylori is an important cause of chronic gastritis leading to peptic ulcer and is a major risk factor for gastric malignancies. Failure in the eradication of H. pylori infection and increasing antibiotic resistance are two major problems in preventing H. pylori colonization. Hence, a deeper understanding of the bacterial survival strategies is needed to tackle the increasing burden of H. pylori infection by an appropriate intervention. Our study demonstrated that the lack of cholesteryl glucosides (CGs) remarkably altered the morphology of H. pylori and increased permeability of the bacterial cell wall. Further, this study highlighted the substantial role of CGs in maintaining the typical H. pylori morphology that is essential for retaining its pathogenic potential. We also demonstrated that the loss of CGs in H. pylori renders the bacterium susceptible to different antibiotics.

## INTRODUCTION

Helicobacter pylori is a highly prevalent human pathogen that colonizes more than 50% of the world’s population. The infection generally results in acute or chronic gastritis and progresses to more severe outcomes such as peptic ulcer disease, mucosa-associated lymphoid tissue (MALT) lymphoma, and gastric adenocarcinoma (1). Amidst global distribution of H. pylori, it is generally held that smart bacterial strategies might contribute to the adaptation of this bacterium to its preferred host (2).

H. pylori has through the course of its evolution and adaptation resorted to a number of strategies to establish persistent infections within gastric and duodenal niches and to evade the host immune system (3). Apart from molecular strategies, some of the structural features, such as the helical shape of the bacilli, which has been suggested to provide a mechanical convenience for penetrating the viscous mucous layer of the stomach, aid in its pathological prowess (4). Another strategy employed by H. pylori for immune evasion is glucosylation of exogenous cholesterol in order to evade the host immune system (5).

Although H. pylori cannot synthesize sterols, the bacteria have the ability to utilize exogenous cholesterol from the living vicinity. It is known that pathogens, such as Mycobacterium tuberculosis, can utilize cholesterol as an energy source (6), while other organisms, such as Borrelia burgdorferi and *Mycoplasma* sp. have the ability to incorporate exogenous cholesterol from their environment and convert it into glycolipids to incorporate into their cell membranes (7, 8). Similarly, H. pylori absorbs cholesterol from host epithelial cells, as it assimilates the secreted lipid and then carries out glucosylation of the exogenous cholesterol to produce three components of cholesteryl glucosides (CGs), cholesteryl-α-D-glucopyranoside, cholesteryl-6-O-tetradecanoyl-α-D-glucopyranoside, and cholesteryl-6-O-phosphatidyl-α-D-glucopyranoside, which is a characteristic feature of H. pylori (9). The glucosylation of cholesterol into CGs in H. pylori is mediated by the enzyme cholesterol-α-glucosyltransferase (CGT) which is encoded by the gene *hp0421*, and deletion of this gene results in the loss of all three CGs (10). CGT is primarily synthesized in cytoplasm in an inactive form and becomes activated when it is bound to the cell membrane (11). Previously, it has been reported that CG content varies in H. pylori when it undergoes morphological changes from the spiral to coccoid form (12).

Morphological alterations in H. pylori were reported upon deletion of cell shape determinants (*csd*) such as peptidoglycan endopeptidase genes, *csd1* and *csd3.* Mutation of these two genes results in rod-shaped and “c”-shaped cells (13). Interestingly, Hildebrandt and McGee observed that the *Hp26695* strain grown in the absence of cholesterol develops an aberrant LPS (14). There are several genes reported to be involved in synthesis of LPS in H. pylori, such as *wecA* and *wzk*, which particularly play essential roles in the synthesis of LPS O-antigens (15). Furthermore, H. pylori grown in the absence of cholesterol showed susceptibility to certain antibiotics, bile salts, and ceragenins (16, 17).

In this study, we investigated the underlying changes in cell morphology, cell integrity, and antimicrobial susceptibility upon deletion of *hp0421* in H. pylori. Our data provide evidence for the loss of typical H. pylori morphology, increase in cell wall permeability, increased sensitivity to antibiotics, and an altered O-antigen expression profile upon deletion of *hp0421*. These findings suggest that loss of cholesteryl glucosides in H. pylori impairs the normal morphology, physiology, and virulence of H. pylori.

## RESULTS

### Loss of cholesteryl-α-glucosides distorts H. pylori morphology.

We consistently observed morphological changes of H. pylori upon deletion of *hp0421* or by cholesterol depletion in growth media.

**FIG 1:**
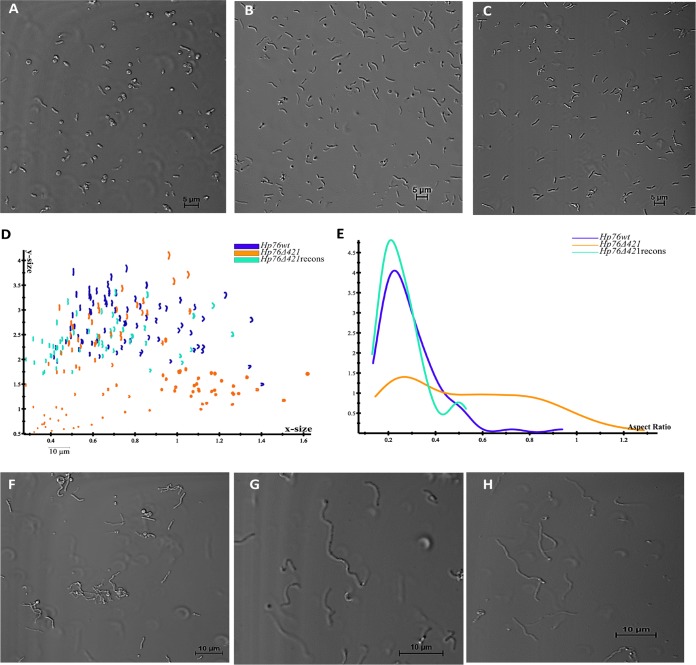
Deletion of the *hp0421* gene perturbs H. pylori cell morphology. (A to C) Confocal microscopy profiles depicting morphological patterns of the *Hp76Δ421* (A), *Hp76* (B), and *Hp76Δ421*-reconstituted (C) strains. (D) Scatter plots arraying *Hp76wt*, *Hp76Δ421*, and *Hp76Δ421*-reconstituted cells (recons) based on *x*-size and *y*-size analyses performed using CellTool software. (E) CellTool analysis output based on plots representing the distribution of *Hp76wt*, *Hp76Δ421*, and *Hp76Δ421*-reconstituted cell populations (recons) according to the aspect ratio. (F to H) Confocal microscopy images for *Hp76Δ421* (F), *Hp76* (G), and *Hp76Δ421*-reconstituted (H) cells when the cells were treated with the filamenting drug aztreonam.

To investigate the effect of CGs on H. pylori morphology, the wild-type (*Hp76*) and knockout (*Hp76Δ421*) strain morphologies were visualized with a confocal microscope by employing transmitted light. The *Hp76Δ421* strain indeed exhibited morphological deformities wherein most cells displayed the coiled “c”-shaped form along with coccoid and rod-shaped bacteria (Fig. 1A), whereas the *Hp76* strain exhibited the normal helical shape (Fig. 1B). Consequently, the reconstitution of the *Hp76Δ421* strain resulted in the recovery of the cell morphology (Fig. 1C). The *Hp26695* strain with or without cholesterol supplementation revealed remarkable variation in the morphology of H. pylori cells (see Fig. S1A in the supplemental material). The altered shapes that were observed included “c” shapes, rods, and coccoid forms in contrast to the normal helical shape observed in bacteria grown in the presence of cholesterol (Fig. S1B).

10.1128/mBio.01523-18.1 FIG S1 Morphology of H. pylori in the absence of cholesterol in culture and upon deletion of *hp0421*. (A and B) Confocal microscopy images depicting H. pylori grown in the absence (A) and presence (B) of cholesterol. (C) Scatter plots showing *Hp26695* and *Hp26695Δ421* cell populations by *x* size and *y* size as analyzed by CellTool. (D) Graphical profiles representative of the distribution of *Hp26695* and *Hp26695Δ421* cell populations according to the aspect ratio and as analyzed by CellTool are shown. (E and F) RNA isolated from and the relative mRNA expression analyses of log-phase cultures of *Hp26695* and *Hp76* strains as quantified by qRT-PCR of *csd1* and *csd3* gene loci (ns denotes nonsignificant differences at *P ≥ *0.05). Download FIG S1, TIF file, 2.1 MB. Copyright © 2018 Qaria et al. 2018 Qaria et al. This content is distributed under the terms of the Creative Commons Attribution 4.0 International license.

Furthermore, we performed quantitative morphological analysis of confocal microscopy images of wild-type (*Hp76*), knockout (*Hp76Δ421*) and *Hp76Δ421*-reconstituted strains using the CellTool software package. It was revealed that the distribution of cell populations of the *Hp76Δ421* strain was bimodal as a result of variable cell shapes, whereas the *Hp76* population distribution was narrower, due to the consistency of cell shapes (Fig. 1D). Moreover, the aspect ratio of cell populations presented significantly uneven distribution due to inconsistencies in the shape of *Hp76Δ421* cells compared to the wild-type cells which demonstrated normal distribution for the aspect ratio (Fig. 1E). Moreover, the reconstitution of *Hp76Δ421* resulted in a wild-type-like cell population distribution. To ensure that the morphological alteration was not dependent on the H. pylori strain used, a similar analysis was performed on *Hp26695* strains. The results of morphology analysis of *Hp26695* and *Hp26695Δ421* strains were in concordance with the results obtained for *Hp76* strains (Fig. S1C and D).

In order to manifest the morphological changes of *Hp76, Hp76Δ421*, and *Hp76*Δ*421* reconstituted strains, they were grown in the presence of aztreonam, an antibiotic that induces pronounced filamentation in H. pylori by inhibiting septal peptidoglycan synthesis. Under these conditions, the *Hp76Δ421* strain exhibited morphological changes such as loss of curvature and stunted filamentation (Fig. 1F) compared to wild-type cells, which exhibited typical curvature and elongated filaments (Fig. 1G). The *Hp76Δ421* reconstituted strain restored the wild-type-like morphologies (Fig. 1H). Moreover, to confirm that the deletion of *hp0421* did not interfere with *csd1* and *csd3* expression, we analyzed their mRNA expression and found that there was no effect on the gene expression levels of *csd1* and *csd3* (Fig. S1E and F). Overall, lack of CGs remarkably impaired the morphology of H. pylori.

### The morphological changes following CG’s absence are attributed to a “c”-shaped bacterial population.

Flow cytometry analysis appears to be an efficient and rapid technique to detect the cell shape of H. pylori at the population level (18). As H. pylori is dependent on exogenous cholesterol for synthesis of CGs, the *Hp26695* strain was grown under microaerophilic conditions with and without cholesterol to analyze the morphological changes by flow cytometry.

**FIG 2:**
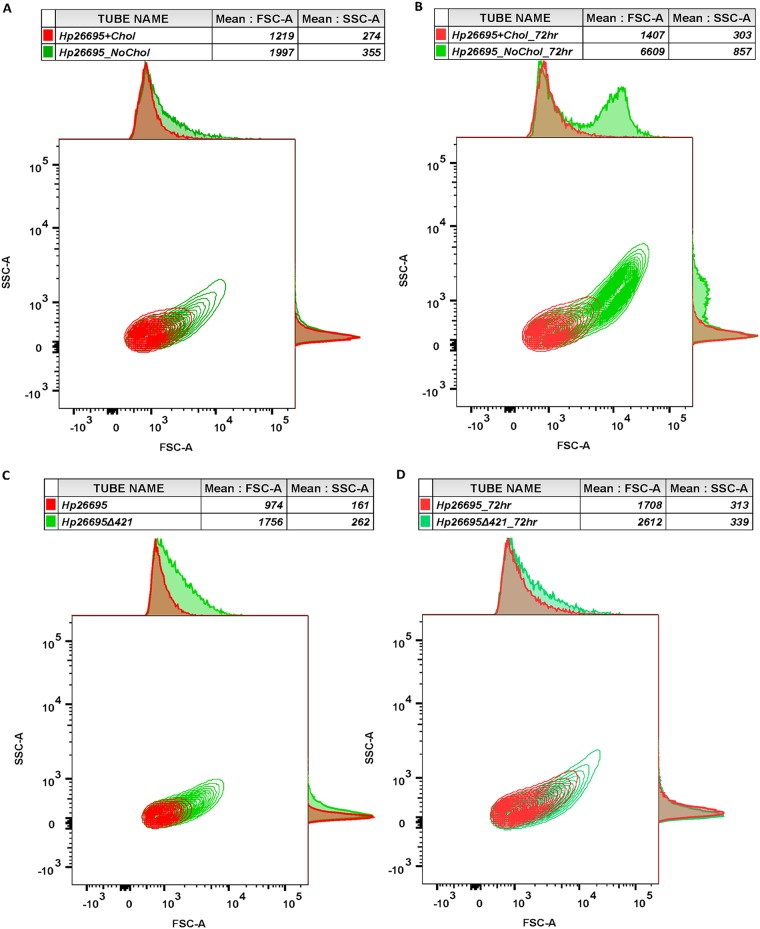
Flow cytometry analysis of H. pylori cell populations based on their shape. Representative contour plots of H. pylori populations depicted based on flow cytometry analyses. Forward scatter (FSC) plotted on the *x* axis and side scatter (SSC) on the *y* axis are presented with mean. (A and B) The *Hp26695* strain was grown in the presence and absence of cholesterol for 48 h (A) and 72 h (B). (C and D) The *Hp26695* and *Hp26695Δ421* strains were grown for 48 h (C) and 72 h (D).

We observed that the *Hp26695* strain grown in the absence of cholesterol exhibited much higher forward scatter (FSC) due to the increased bulk width of the bacterial cell, which represents “c”-shaped cells compared to the cells grown in cholesterol-containing medium. Moreover, *Hp26695* cells grown in the absence of cholesterol have displayed slightly higher side scatter (SSC) value, which indicates that cells presented higher granularity or complexity (Fig. 2A). Notably, the *Hp26695* strain grown for 72 h in the absence of cholesterol also displayed remarkably higher FSC and SSC values compared to the strain grown only for 48 h (Fig. 2B). In line with the above results, the *Hp26695*Δ*421* strain also displayed higher FSC and SSC values in both 48 h and 72 h culture populations compared to the wild-type strain (Fig. 2C and D). Furthermore, the population shape outcomes of *Hp76* and *Hp76*Δ*421* strains as analyzed by flow cytometry were in accordance with the observation recorded for *Hp26695* and *Hp26695Δ421* strains, and interestingly, the cell wall integrity was restored in *Hp76*Δ*421* reconstituted strain (Fig. S2A and B).

10.1128/mBio.01523-18.2 FIG S2 Morphology of *Hp76* strains presented by flow cytometry. Representative contour plots of H. pylori populations when analyzed by flow cytometry; FSC and SSC are shown on the *x* and *y* axes, respectively, with mean and median. *Hp76Δ421* displayed high FSC and SSC compared to the *Hp76* and *Hp76Δ421*-reconstituted strains grown for 48 h (A) and 72 h (B). Download FIG S2, TIF file, 0.5 MB. Copyright © 2018 Qaria et al. 2018 Qaria et al. This content is distributed under the terms of the Creative Commons Attribution 4.0 International license.

Taken together, these observations indicate that the changes in morphology of the *Hp26695Δ421* and *Hp76Δ421* strains or of wild-type strains grown in the absence of cholesterol were due to the presence of “c”-shaped cell population, which is evidenced by the higher FSC values.

### Deletion of *hp0421* results in cell wall fluidity.

**FIG 3:**
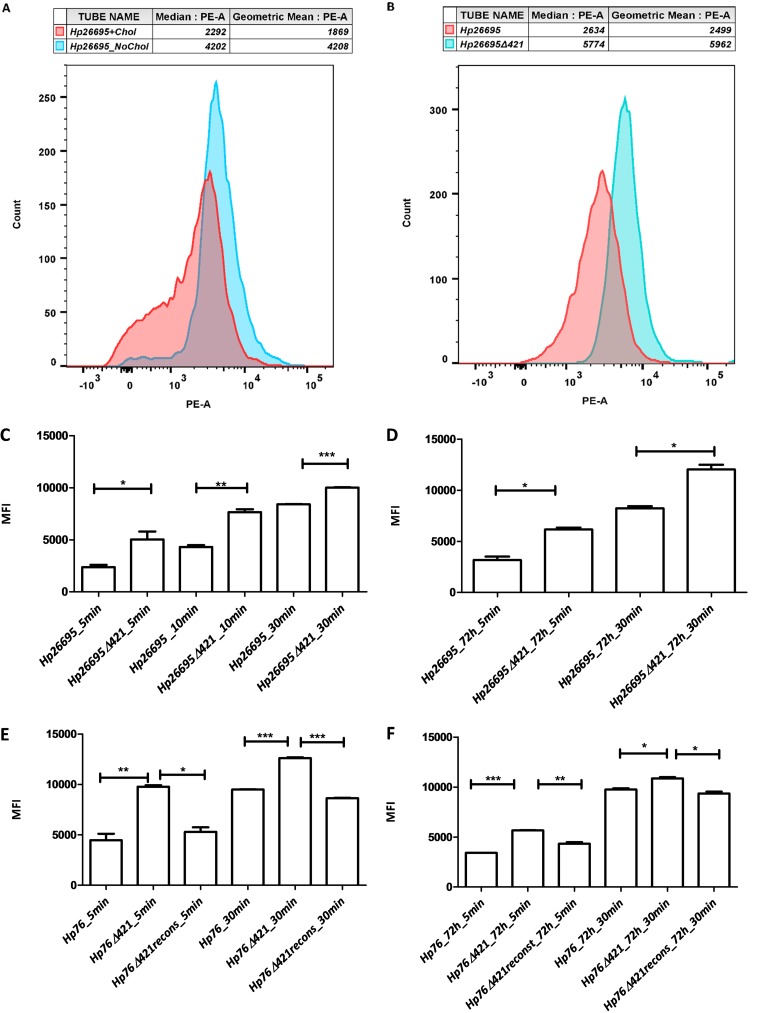
Absence of cholesteryl glucosides increased H. pylori cell wall permeability. (A and B) Flow cytometry analysis based on influx rates of EtBr as depicted in the form of PE-A histograms delineating the median fluorescence intensity (MFI) for *Hp26695* strain grown in the presence and absence of cholesterol (A). (B) Influx rates of EtBr for *Hp26695* and *Hp26695Δ421* strains grown for 48 h. (C to F) The bars represent MFIs for wild-type, knockout, and reconstituted strains (as annotated) when treated/incubated with EtBr for 5, 10, and 30 min using cultures grown either at 48 h or 72 h (*, *P < *0.05; **, *P ≤ *0.01; ***, *P ≤ *0.001).

H. pylori CGs comprise more than 25% of total cell wall lipids (19). Hence, to study whether the depletion of CGs has any effect on H. pylori cell wall permeability, we measured the influx of ethidium bromide (EtBr) among wild-type and knockout strains by flow cytometry. Bacterial strains were incubated with EtBr, and the median fluorescence intensity (MFI) was measured. We observed that the *Hp26695* strain grown in the absence of cholesterol showed higher MFI than the strain grown in the presence of cholesterol (Fig. 3A), indicating a higher influx of EtBr in bacterial culture grown in the absence of cholesterol. We also measured EtBr fluorescence intensity between *Hp26695* and *Hp26695Δ421* strains; the MFI was significantly higher in the *Hp26695Δ421* strain than in the wild type (Fig. 3B). To determine the kinetics of EtBr influx in *Hp26695* and *Hp26695Δ421* strains, we measured the MFI of EtBr influx at 5, 10, and 30 min time intervals. The MFI of the *Hp26695Δ421* strain was significantly higher through all the time intervals compared to the MFI observed for the *Hp26695* strain (*P* value of <0.05) (Fig. 3C).

In order to check the cell wall fluidity of lag-phase cultures, the *Hp26695Δ421* and *Hp26695* strains were cultured for 72 h and were observed for EtBr influx. As expected, the MFI was found to be significantly higher in the *Hp26695Δ421* strain than in the *Hp26695* strain (*P* value of <0.05) (Fig. 3D and Fig. S3A). Furthermore, we observed that the *Hp76*Δ*421* strain exhibited higher MFI compared to *Hp76* and *Hp76*Δ*421* reconstituted strains (48 h culture) (Fig. 3E and Fig. S3B). We also measured the MFI for *Hp76* strains in 72 h cultures and found that the influx rate was significantly higher in the *Hp76Δ421* strain than in the *Hp76* and *Hp76*Δ*421*-reconstituted strains (*P* value of <0.05) (Fig. 3F and Fig. S3C and D). Overall, from these observations, we anticipate that the lack of CGs would likely perturb the cell wall permeability of H. pylori by damaging the cell wall integrity.

10.1128/mBio.01523-18.3 FIG S3 The absence of cholesteryl glucosides caused increased cell wall permeability. **(**A to D**)** PE-A histograms delineating the influx rates of EtBr represented as MFI for different wild-type and knockout strains grown in culture for different periods and when treated with EtBr at different time points as shown. Download FIG S3, TIF file, 1.1 MB. Copyright © 2018 Qaria et al. 2018 Qaria et al. This content is distributed under the terms of the Creative Commons Attribution 4.0 International license.

### Lack of CGs disrupts LPS structure.

**FIG 4:**
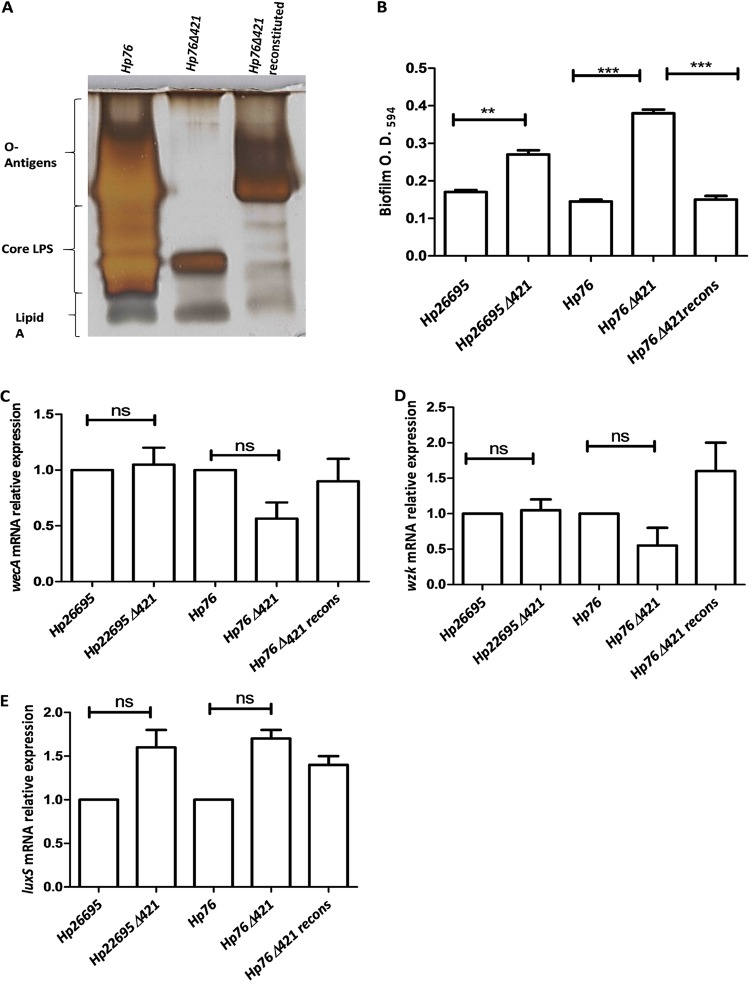
Analysis of LPS expression and biofilm formation. (A) Silver-stained SDS-PAGE gel (15%) depicting profiles of LPS from *Hp76*, *Hp76Δ421,* and *Hp76Δ421*-reconstituted strains. (B) Bar graph representing quantification of biofilm formation by H. pylori after 5 days of incubation. The *Hp26695* strain data were analyzed by Student’s *t* test. The *Hp76* strain data were analyzed by one-way ANOVA followed by Tukey’s multiple-comparison tests. (C and D) qRT-PCR analyses of *wecA* and *wzk* genes using RNA isolated from wild-type (*Hp26695* and *Hp76),* knockout *(Hp26695Δ421* and *Hp76Δ421),* and *Hp76Δ421-*reconstituted strains grown for 48 h. (E) Relative mRNA expression of *luxS* from 2-day-old broth cultures [ns, nonsignificant difference(s); *, *P < *0.05, **, *P ≤ *0.01; ***, *P ≤ *0.001].

In order to detect the changes in O-antigen expression due to disruption of CGs, we isolated lipopolysaccharides from *Hp76*, *Hp76Δ421,* and *Hp76Δ421*-reconstituted strains and visualized them by silver-stained SDS-polyacrylamide gel electrophoresis. Depletion of CGs resulted in the disruption of O-antigens as observed by silver staining of *Hp76Δ421* LPS in which the O-antigens were absent compared to the *Hp76* LPS profile. Consequently, the O-antigens and core LPS were partially restored in the *Hp76Δ421*-reconstituted strain (Fig. 4A). Moreover, we investigated whether the deletion of *hp0421* in *Hp26695* and *Hp76* strains had any effect on the transcription of *wecA* and *wzk* genes. We found no significant differences in the gene expression levels for these two genes among wild-type *H. pylori*, their *hp0421* mutant(s), and the reconstituted strain (Fig. 4C and D). This observation rules out the possibility that the alteration in LPS profile was due to changes in the expression of these O-antigen synthesis genes. Thus, the lack of CGs most likely disrupted the normal components of H. pylori LPS.

### Cholesteryl glucosides’ perturbation in H. pylori renders bacteria sensitive to antibiotics.

To investigate the effect of loss of CGs in H. pylori on antibiotic resistance, we determined the MICs of several antibiotics on *Hp26695* and *Hp76* strains. For this, the strains were grown on Brucella agar plates in the presence of MIC E-strips belonging to different antibiotics. *Hp26695* and *Hp76* strains were found to be resistant to fosfomycin, polymyxin B, colistin, tetracycline, and ciprofloxacin. Interestingly, the *Hp26695Δ421* and *Hp76Δ421* strains, on the other hand, were found to be sensitive to all of the above antibiotics tested (Table 1). Further, the *Hp26695*Δ*421* was more sensitive to amoxicillin than *Hp26695*, while both of them were sensitive to clarithromycin. The increased sensitivity of *Hp26695Δ421* and *Hp76Δ421* strains was understandable, as these strains demonstrated an increase in cell wall permeability and altered LPS profile due to the loss of CGs. Overall, it appears that the deletion of *hp0421* perturbs the cell wall integrity of H. pylori, which in turn leads to increased susceptibility to all the antibiotics tested.

**TABLE 1:** MICs of wild-type, *Δ421* mutants, and *Δ421*-reconstituted H. pylori strains

Antibiotic	MIC (μg/ml) of the following strain(s)^*a*^:				
	*Hp26695*	*Hp26695Δ421*	*Hp76*	*Hp76Δ421*	*Hp76Δ421-* reconstituted
Fosfomycin	≥1,024 (R)	≤1.5 ± 0.5 (S)	≥1,024 (R)	≤0.064 ± 2 (S)	≥1,024 (R)
Colistin	≥256 (R)	≤12 ± 3 (S)	≥256 (R)	≤10 ± 5 (S)	≥256 (R)
Polymyxin B	≥256 (R)	≤8 ± 4 (S)	≥256 (R)	≤10 ± 4 (S)	≥256 (R)
Ciprofloxacin	≤0.38 ± 0.5 (R)	≤0.064 ± 0.02 (S)	≤0.50 ± 0.5 (R)	≤0.19 ± 0.2 (S)	≤0.047 ± 0.08 (R)
Tetracycline	≤10 ± 3 (R)	≤0.01 ± 0.02 (S)	≤0.50 ± 0.1 (R)	≤0.01 ± 0.09 (S)	≤0.38 ± 0.18 (R)
Amoxicillin	≤0.47 ± 0.01 (R)	≤0.016 ± 0.01 (S)	≤0.016 ± 0.01 (S)	≤0.016 ± 0.01 (S)	≤0.016 ± 0.01 (S)
Clarithromycin	≤0.001 (S)	≤0.001 (S)	≤0.001 (S)	≤0.001 (S)	≤0.001 (S)

a The letters in parentheses after the MICs indicate whether the strain is resistant (R) or sensitive (S) to the respective antibiotic.

### Deletion of *hp0421* enhances H. pylori cell aggregation.

H. pylori produce biofilms on stomach mucosa in order to circumvent the gut’s harsh environment, and it was reported that the deletion of *luxS* gene increases the biofilm formation (20). We observed that *Hp26695Δ421* and *Hp76Δ421* strains tended to aggregate in the liquid cultures in contrast to the wild-type cultures that appeared turbid in their growth. Therefore, we investigated the effect of *hp0421* deletion on biofilm formation in *Hp26695* and *Hp76* strains. Biofilm formation was visualized on glass coverslips under the microscope. The *Hp26695Δ421* and *Hp76Δ421* strains demonstrated biofilm formation on the third day, while the wild-type strain formed biofilm on the fifth day. Moreover, crystal violet absorbance assay revealed that the biofilms formed by *Hp26695Δ421* and *Hp76Δ421* strains were significantly denser than the biofilms formed by the *Hp26695*, *Hp76*, and *Hp76Δ421*-reconstituted strains (Fig. 4B). The results showed that the *Hp26695Δ421* and *Hp76Δ421* strains tended to aggregate and adhere strongly to the surface of the coverslip at the air-liquid interface. Hence, perhaps, the lack of CGs enhances aggregation of bacterial cells and promotes biofilm formation in H. pylori. Moreover, we determined the expression of the *luxS* gene in the above strains to check whether its expression is affected by the deletion of *hp0421*. We found no significant differences in the gene expression levels for *luxS* in both wild-type and *Hp26695Δ421* and *Hp76Δ421* strains (Fig. 4E).

## DISCUSSION

In the present study, we analyzed the possible role of CGs in H. pylori in the maintenance of normal spiral shaped bacillary morphology and cell wall integrity and investigated their role in sensitivity and resistance to antibiotics. We also studied the effect of loss of CGs on lipopolysaccharide profiles and on biofilm formation using *Hp26695Δ421* and *Hp76Δ421* (knockout) strains and their respective wild types. We observed the presence of CGs to be essential for maintaining the normal, spiral morphology of H. pylori, while the lack of CGs remarkably distorted the shape of H. pylori cells to variable structures, with coiled and “c”-shaped cells being dominant. A previous study also observed similar morphological alterations upon deletion of peptidoglycan endopeptidase genes *csd1* and *csd3* in H. pylori (13). However, we found that there was no effect on the gene expression levels of *csd1* and *csd3* upon deletion of the *hp0421* gene. Moreover, CGs are the major constituents of the H. pylori cell wall and comprise more than 25% of the total cell wall lipids (19). Given this, our results are strongly suggestive of CG depletion leading to alteration of lipid raft components of the H. pylori cell wall which could have disrupted its integrity. Since lipid rafts are required in maintaining the architecture of the cell wall and order of cell wall domains, absence of the CGs may be linked to change in the normal helical shape of H. pylori. In fact, a study on Borrelia burgdorferi wherein cell wall cholesterol depletion by methyl-β-cyclodextrins (MβCD) without substitution by other sterols resulted into coiled spirochetes (21). Our results are in line with this observation which supports that the lack of sterols alters the morphology of H. pylori. Moreover, the results of analysis of H. pylori morphology suggest that the lack of CGs remarkably altered the size and curvature of the cells; these results strongly point to the possibility that CGs are part of lipid rafts in the cell wall and that they play a crucial role in maintaining the typical helical shape and size of H. pylori cells. It should be noted that the helical shape of H. pylori is a major factor in the process of invasion of gastric niches. This is of particular significance given the reports that the colonization rates in mice stomach by the helical rod-shaped H. pylori were higher than those of the *csd1* and *csd3* mutants that were curved and rod shaped (13). Similarly, Wunder et al. reported that *hp0421* mutant(s) failed to colonize C57BL/6 mice and were cleared from the gastric tissue (5). These results demonstrate that the changes in H. pylori morphology due to the absence of CGs negatively influence the colonization potential of H. pylori. We speculate that this could be a result of direct or indirect interaction between the CGs and the peptidoglycans that altered the order of cell wall domains.

The deletion of the *hp0421* gene results in depletion of CGs in the cell wall, which could lead to enhanced permeability. Likewise, when H. pylori was grown in the absence of cholesterol, bacterial cell permeability was significantly increased. Thus, this indicates the critical role of CGs in the formation of ordered cell wall units of H. pylori to maintain its integrity. In an alternative scenario, the CGs are probably required to maintain the tight pack of the outer wall of H. pylori. Sterols in the membrane are believed to support the cell membrane in B. burgdorferi, and the depletion of membrane cholesterol and substitution with different sterol analogues increased the permeability of the membrane at different levels based on the type of sterols substituted. The depletion of cholesterol by MβCD without substitution with any sterols caused significant increase in the permeability of the membrane (21). Also, in yeast, the deletion of essential genes of sterol biosynthesis was reported to increase the cell membrane permeability (22). Our findings are in line with these and other reports and suggest that CGs interact with peptidoglycan domains in order to maintain the architecture of the H. pylori cell wall. The permeabilization of H. pylori cell wall has far-reaching implications for therapeutic interventions.

Further, we observed that the *Hp76Δ421* strain exhibited aberrant LPS expression profile with loss of O-antigens and lack of core LPS. Our observations suggest that the perturbation of the architecture of the H. pylori cell wall due to the lack of CGs reduced LPS expression. On the other hand, this might have occurred due to the changes in the structure of the cell wall affecting the O-antigen biosynthesis enzymes that are present in the cell wall. It is relevant in this context that the deletion of *hp0421* did not affect the mRNA expression of essential LPS synthesis genes *wecA* and *wzk*. Alternatively, the changes in the structure and composition of the cell wall due to the lack of CGs may result in the dysregulation of the transfer of LPS units through membranes. Our observations appear to be in agreement with the report of Hildebrandt et al., who observed that depletion of cholesterol leads to the development of aberrant LPS in the *Hp26695* strain, which depends on lipid A phosphorylation (14).

Furthermore, we observed that lack of CGs rendered H. pylori sensitive to antibiotics. The increased susceptibility to the antibiotics due to the deletion of *hp0421* may be the result of permeabilization of the cell wall, facilitating antibiotics to penetrate passively through H. pylori cells. The bacterial cell wall is the first line of defense against antibiotics, detergents, and host defense elements. The membrane/cell wall permeabilization could be an effective method to control bacterial infections by enhancing antibiotic action/delivery (23). Second, we suggest that disruption of LPS and/or influencing the outer membrane charge due to the lack of CGs decreased the resistance of H. pylori to certain antibiotics like polymyxin and colistin. Disruption of *lpx_EHP,_* a gene encoding lipid A in H. pylori, has been shown to dramatically decrease the polymyxin resistance from a MIC of >250 µg/ml to a MIC of 10 µg/ml (24). Similarly, we believe that the deletion of *hp0421* might possibly affect the LPS structure thus influencing the outer membrane charge and cell wall integrity and eventually increasing the sensitivity of H. pylori to certain antibiotics. Inhibition of CG synthesis may not kill the bacteria directly but rather render the pathogens incapable of establishing successful infection by hindering their colonization potential and fitness advantage (resistance toward antibiotics).

Moreover, we suggest that the tendency of *Hp26695Δ421* and *Hp76Δ421* strains to aggregate on the surface of coverslips is probably due to changes in the properties of LPS. Modifications in LPS have been shown to enhance bacterial autoaggregation and biofilm formation (25). Alternatively, the changes in the cell wall properties and morphology may trigger stress-related genes, including the quorum-sensing gene *luxS.* Similarly, the changes in the membrane structure of Pseudomonas aeruginosa were previously reported to alter quorum sensing (26). However, we did not observe any changes in the mRNA expression levels of *luxS*.

In conclusion, we have shown the role of CGs in the maintenance of H. pylori’s native helical morphology, and the lack of CGs remarkably resulted in variable shape and size of H. pylori cells dominated by “c”-shaped cells. Moreover, the cell wall permeability increased irrespective of the duration of culture and the strain type of H. pylori. Further, we showed that lack of CGs perturbed the structure of cell wall components like LPS, which on one hand, would attenuate the virulence of H. pylori and, on the other hand, would render H. pylori susceptible to antibiotics to which it was otherwise resistant. CGs could be a promising target for drugs aiming at the eradication of H. pylori infection. The functional roles of CGs in H. pylori that we report here significantly extends the previous understanding on the role of cholesterol glucosylation in H. pylori immune evasion and pathogenicity. Future studies are needed to determine whether inhibition of H. pylori-specific CGs constitutes a target for the development of new therapeutic molecules for H. pylori-induced inflammation and malignancies.

## MATERIALS AND METHODS

### Bacterial strains and cholesterol loading.

The human-adapted *Hp26695* strain and its mutant strain, the *Hp26695Δ421* strain, were grown on GC agar medium (Difco, USA) supplemented with 10% horse serum, 2.5 µg/ml trimethoprim, 10 µg/ml vancomycin, and 1 µg/ml nystatin as described previously (27) (antibiotics were excluded in experiments of antibiotic resistance determination and aztreonam filamentation assay). An aliquot of 4 µg/ml kanamycin was added as a resistance selection marker for *Hp26695Δ421*. The mouse-adapted *Hp76* strain, its mutant strain (the *Hp76Δ421* strain), and the reconstituted *Hp76Δ421* strain were kindly provided by Thomas F. Meyer, Max-Planck Institute for Infection Biology, Germany. *Hp76* strains were cultured as described previously (5). Further, the procedures to grow Helicobacter pylori in the absence of cholesterol were modified from earlier described method(s) (28). Briefly, the wild-type strains were grown on Ham’s F-12 medium (Gibco, USA) (chemically defined) supplemented with 1 mg/ml BSA with or without 1 mM water-soluble cholesterol (250 µM cholesterol with 4 mM MβCD) (Sigma). For agar plates, Ham’s F-12 medium was prepared (2×) and mixed with 30 g per liter agar at 1:1 ratio. For broth culture, the strains were grown in Brucella broth medium with the use of CampyGen compact sachets (Oxoid, UK) inside a shaker-incubator to create a microaerophilic condition. To visualize cell elongation, 2 µg/ml of aztreonam antibiotic was added to the medium. All strains were incubated under humidified microaerophilic conditions with 5% O_2_ and 5% CO_2_. All wild-type and mutant strains of H. pylori were handled as per biological safety level-2 norms with required permissions.

### Generation of *hp0421* mutant in *Hp26695* strain.

The cholesterol α-glucosyl transferase *hp0421* (Gene ID 900074) knockout in *Hp26695* was generated by homologous recombination as described previously (10). Briefly, two pairs of primers upstream (PCR1) and downstream (PCR2) of *hp0421* regions were designed with XhoI restriction enzyme sequence (Table 2). Ligated PCR1 and PCR2 were inserted by TA cloning into the pTZ57R/T plasmid, followed by transformation into Escherichia coli DH5α. The plasmids were purified by Plasmid Miniprep kit (Qiagen, Germany) and digested with XhoI. The kanamycin resistance cassette was inserted between PCR1 and PCR2 and subsequently cloned into E. coli DH5α. The purified plasmids were transformed into the *Hp26695* strain by natural transformation. The transformed bacteria were grown on GC agar kanamycin medium. Additionally, the absence of the pTZ57R/T plasmid was confirmed by sensitivity to ampicillin, and the presence of the knockout construct was confirmed by PCR and construct sequence analysis.

**TABLE 2:** Primers used in this study

Target gene of the primer	Nucleotide sequence of the primer
*hp0421* US F	GTGGATTATGACTCTTTAGAGACTTG
*hp0421* US R	GTGCCATGGCTCGAGTTAACTACTCTTCTTTAAAATTGAAT
*hp0421* DS F	GTGCCATGGCTCGAGTGAAAGGATAAAAAATGCAAGAA
*hp0421* DS R	CCAATTTTAGGGCAGGCTAAAAAC
*wecA* F	ATGGTGCTTGGGTTTATGGTG
*wecA* R	GGCTTTCTGGCGTTTTATTTTG
*wzk* F	AAACTCAAAGACAACCACGAAG
*wzk* R	CGACCGCTAAAATCAACAAG
16s rRNA F	GGTAAAATCCGTAGAGATCAAGAGG
16s rRNA R	ACAACTAGCATCCATCGTTTAGG
*cds1* F	GGATGAATTTTTAGACGATTTGC
*cds1* R	CCCTCTTCTTTTTCTTCTTCAGG
*cds3* F	CTAAACATGGCAGCTTGATCC
*cds3* R	AATGGATTTCAACCACCTTCC
*luxS* F	TTTGATTGTCAAATACGATGTGC
*luxS* R	TGTGAGATAAAATCCCGTTTGG

### Cell wall fluidity and H. pylori cell morphology by flow cytometry.

For cholesterol depletion-based selection, the *Hp26695* strain was grown on chemically defined medium. The wild-type *Hp26695*, *Hp26695Δ421*, wild-type *Hp76*, *Hp76Δ421*, and *Hp76Δ421*-reconstituted strain were all grown for 48 to 72 h on Brucella agar (BD Biosciences). The staining and measurement procedures for EtBr influx were modified from a previous study (29). Briefly, bacterial cells were collected and washed thrice with PBS at pH 7.4 (Gibco, USA). A total of 10^6^ cells were resuspended in 1 ml PBS or in 1 ml PBS (containing 5 µg of EtBr filtered through a 0.22-µm Millix-GV syringe filter [Merck-Millipore, USA]). All tubes were incubated at 37°C with gentle mixing inside a hybridization rotor for 5 to 30 min, followed by flow cytometry analysis on a BD FACS Canto II system (BD Biosciences). EtBr fluorescence intensity was measured by flow cytometry analysis with the excitation wavelength set at 488 nm and the fluorescence emission set at 585 nm. The data were analyzed by using FlowJo LLC software.

### H. pylori morphology analysis upon deletion of *hp0421*.

H. pylori cultures grown for 48 h were washed by PBS (pH 7.0) with 10% glycerol thrice, and the cells were adjusted to an OD_550_ of 0.2. Cells were mounted on glass slides and imaged by confocal microscope (model LSM 880; Carl Zeiss). The image optimization was carried out in Adobe Photoshop 7. The quantitative analysis of processed images to measure the *x* size, *y* size, and aspect ratio of H. pylori cells were done individually by CellTool software package as described previously (30).

### Quantitative PCR and gene expression analysis.

The method we followed for qRT-PCR analysis was described previously (31). Briefly, RNA was isolated from 10^8^ cells of H. pylori strains by TRIzol (Invitrogen, USA). Subsequently, 3 µg of RNA was converted to cDNA using SuperScript-III (Invitrogen, USA) and random hexamers according to the manufacturer’s instructions. For qRT-PCR, 40 ng of the first transcribed DNA strand was amplified by using SYBR Fast qPCR Mix (TaKaRa, Japan) with primers targeting *cds3*, *cds1*, *wzx*, and *wecA* genes and 16S rRNA as an internal control. For *luxS*, H. pylori strains were grown in Brucella broth for 2 days, and RNA was isolated from planktonic and sessile bacterial cells. The primer sequences are listed in Table 2.

### Determination of antibiotic resistance among wild-type H. pylori and *hp0421* mutant.

*Hp26695* and *Hp76* strains grown on brain heart infusion agar medium were collected and washed with PBS. About 50 µl of 10^8^ H. pylori cell suspension was spread on brain heart infusion agar medium and antibiotic-impregnated strips (HiMedia, India) corresponding to clarithromycin (0.016 to 256 μg/ml), amoxicillin (0.016 to 256 μg/ml), fosfomycin (0.064 to 1024 μg/ml), polymyxin B (0.016 to 256 μg/ml), colistin (0.016 to 256 μg/ml), tetracycline (0.016 to 256 μg/ml), and ciprofloxacin (0.002 to 31 μg/ml) were placed on the plates and incubated for 3 days. The susceptibility was defined by breakpoints defined by the Clinical and Laboratory Standards Institute (CLSI) (32).

### Biofilm formation by H. pylori *hp0421* mutant strains.

Biofilm formation was assayed using a modified protocol as described previously (33, 34). Briefly, *Hp26695* and *Hp76* cells were collected from BHI agar and washed with PBS. Inocula at an OD_550_ of 0.2 were seeded in 12-well plates, each well contained 2 ml of Brucella broth with 7% decomplemented horse serum (Gibco, USA), and sterilized glass coverslips were used to cover the wells to allow adherence of H. pylori at the air-liquid interface. The cultures were incubated under microaerophilic conditions at 37°C for 2 to 6 days. After incubation, the coverslips were washed with PBS, followed by staining with 0.1% crystal violet stain. The coverslips were further rinsed with PBS and dried. The associated dye was dissolved in acetone and ethanol (2:8), and the absorbance was measured by microplate reader at 594 nm.

### Lipopolysaccharide purification and visualization.

Purification of lipopolysaccharides from H. pylori strains was carried out according to the previously described method of Hong et al. with slight modifications (35). Briefly, the bacterial lawns were collected and washed with 1 ml PBS (pH 7.4), followed by centrifugation thrice at 10,000 rpm for 10 min in each case. The pellets were resuspended in lysis buffer (60 mM Tris-HCl [pH 6.8], 2% SDS) and incubated at 98°C for 10 min, and the whole-lysate protein was quantified by BCA (bicinchoninic acid assay) (Thermo Fisher Scientific, USA). LPS was extracted by adding 45% hot phenol to the lysate, vortexed vigorously, and incubated at 70°C for 30 min. The mixtures were centrifuged at 16,000 × *g* for 15 min, and the upper phase layer was collected in 2-ml tubes and LPS was precipitated by adding 75% cold ethanol and 10 mM sodium acetate. The tubes were then incubated at −20°C overnight, followed by centrifugation at 16,000 × *g* for 15 min. To remove DNA and RNA contaminants, 3 µl of buffer 2 (NEB, UK), 0.5 mg/ml DNase I (amplification grade) (Sigma, USA), and 0.5 mg/ml RNase A (Invitrogen, USA) were added, followed by incubation for 1 h at 37°C and treatment with 0.5 mg/ml proteinase K (Amresco, USA) for 1 h at 56°C. The LPS was re-extracted by adding 50% phenol, followed by vigorous vortexing and centrifugation. The pellet was finally precipitated by cold ethanol, resuspended in 50 µl of deionized water, and stored at −80°C. For visualization of LPS, 10 µl from each tube was loaded on a 15% SDS gel and stained with dual silver stain (36). The LPS units were quantified by *Limulus* amebocyte lysate (LAL) chromogenic endotoxin quantitation kit (Pierce, USA) according to the manufacturer’s instructions.

### Statistical analysis.

The statistical analyses were performed using Student’s *t* test and one-way ANOVA followed by Tukey’s multiple-comparison tests. The data are presented as mean ± the standard error (of mean) from three independent experiments.
