# Supplementary figures and images for: Roles of Cholesteryl-α-Glucoside Transferase and Cholesteryl Glucosides in Maintenance of Helicobacter pylori Morphology, Cell Wall Integrity, and Resistance to Antibiotics
Source: mBio. 2018 Nov 27;9(6):e01523-18. doi: 10.1128/mBio.01523-18 (PMC6282200; doi:10.1128/mBio.01523-18)

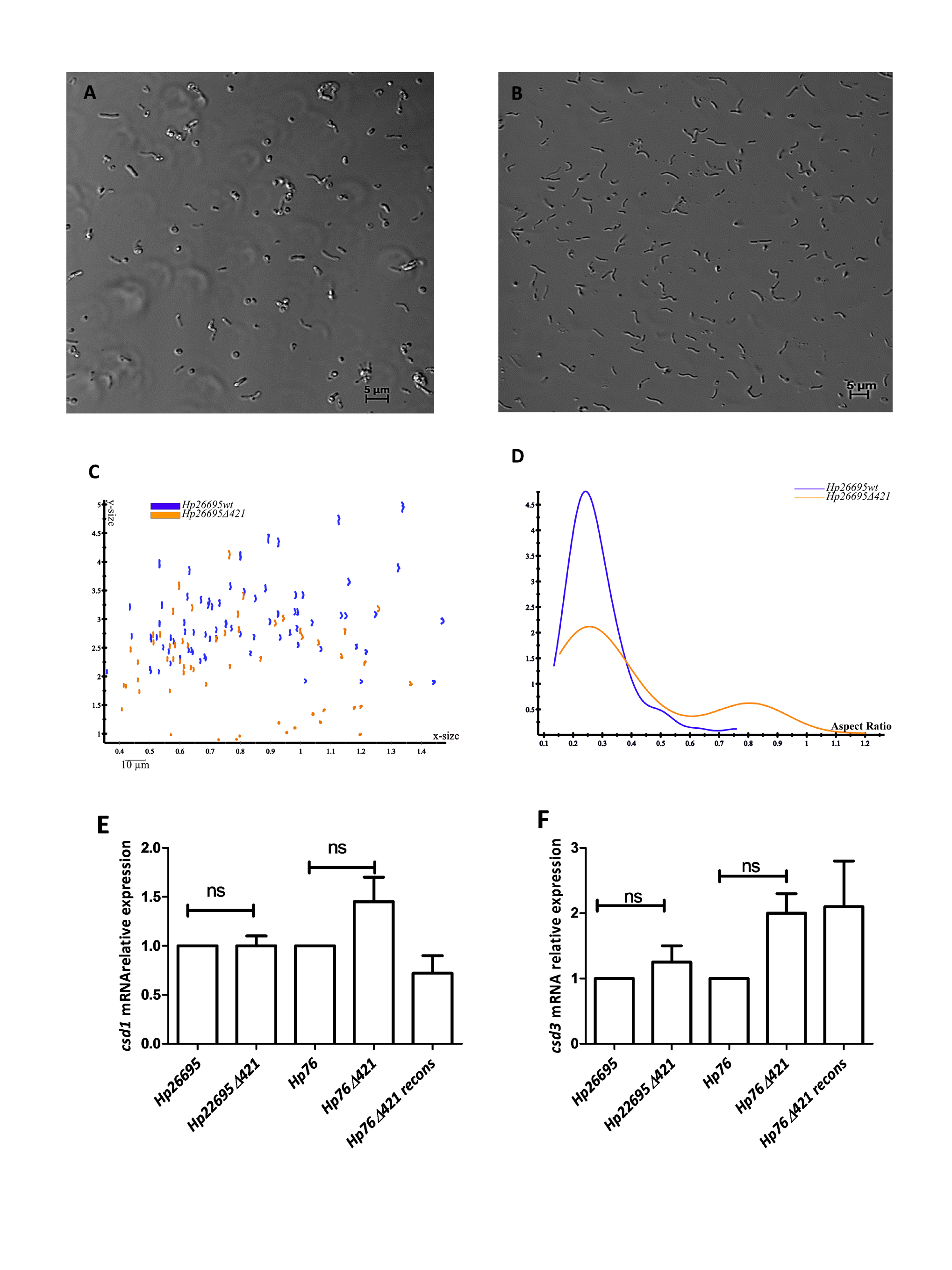

Supplement: FIG S1 [file mbo006184189sf1.tif]

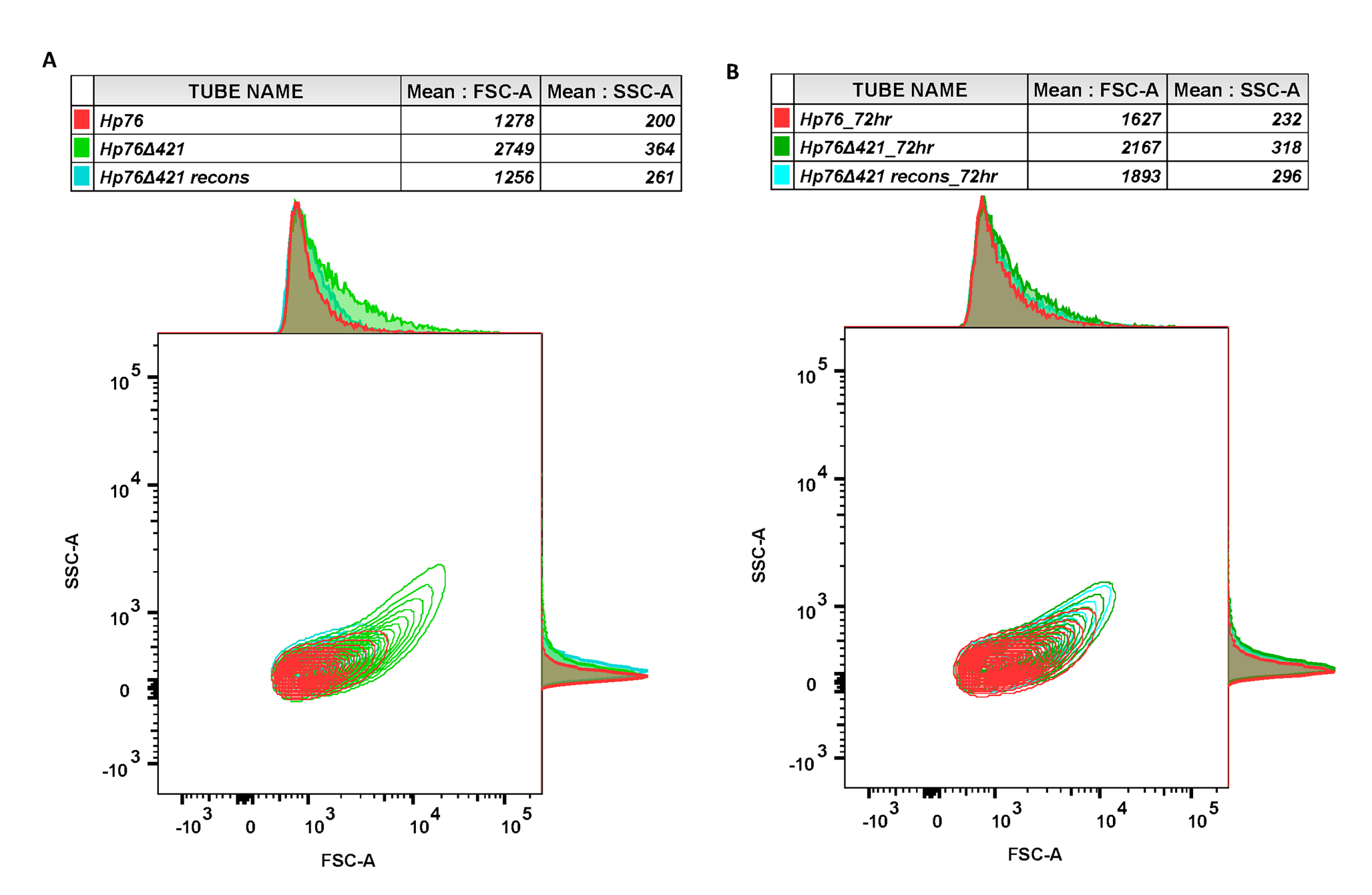

Supplement: FIG S2 [file mbo006184189sf2.tif]

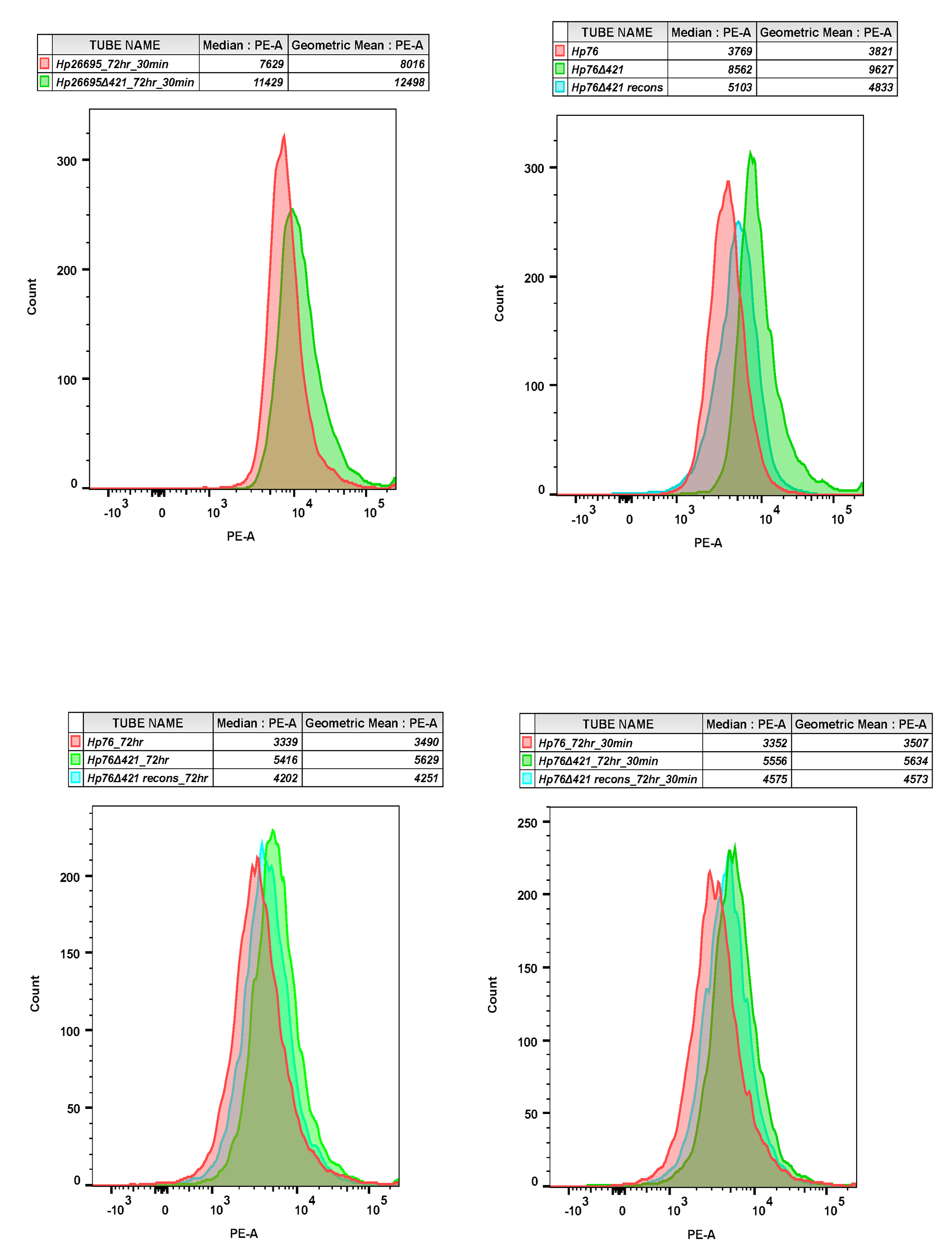

Supplement: FIG S3 [file mbo006184189sf3.tif]
